# Sustainable youth employment quality management: The impact of robotization in China

**DOI:** 10.1371/journal.pone.0298081

**Published:** 2024-04-26

**Authors:** Fucheng Liang, Yi Liu

**Affiliations:** 1 School of Shenzhen Tourism, Jinan University, Shenzhen, Guangdong, China; 2 School of Management, Jinan University, Guangzhou, Guangdong, China; Zhejiang University, CHINA

## Abstract

Robotization has caused widespread concern about job losses, but few scholars have paid attention to changes in employment quality. This study provides supplementary evidences on the impact of robotization on youth employment quality and compares the effectiveness of various measures. Using data about individual employment and robot usage in China, this study finds that robotization reduces youth employment quality, especially for males and the middle-educated, aged 26 to 35, and in regions with insufficient workers. The substitution effect, skill preparation effect, and productivity effect play important roles in this process. Besides the common strategy of education, the mitigating capabilities of skill training has been demonstrated, but self-entrepreneurship has not. This study suggests that the exploration of various youth self-development measures, such as skill training, is warranted to improve employment quality.

## Introduction

In recent years, the related technologies of artificial intelligence, automation, and robotics have developed rapidly worldwide, especially the application of robotics, which has gradually spread from developed countries to developing countries. According to data from the International Federation of Robotics (IFR) report, 71% of industrial robots are deployed in Asia in 2020. While improving work efficiency, robots have also shocked the job market to some extent. Some scholars believe that robots will not only reduce the number of jobs [1–3], but also negatively affect work intensity [4]. This idea is borne out by data from developed countries, but there is little evidence of robotization’s impact in developing countries.

The Asia-Pacific region and other emerging markets have undergone a major industrial transformation in the past decades and differ from developed countries in robot usage, application scenarios, and human trust stages [5]. In addition, as a representative of emerging markets, China faces significant youth employment issues. Even more unfortunately, robotization is likely to reduce lower-level jobs traditionally serving as a “pass-through” for youth transitioning from school to permanent employment in the primary sector [6].

The theory of skills-biased technological change (SBTC) suggests that unemployment risk and income polarization can be alleviated by increasing investment in education [7]. However, this doesn’t explain the phenomenon that young people in developing countries are better educated than in the past, while satisfying jobs are harder to find. This paradox emphasizes a pressing problem in the robotization literature: whether the theory of SBTC is suitable for young people in developing countries? Without answer to this question, the experience in developed countries will be blindly applied to developing countries. If existing theories can’t explain the declining contribution of education, this threatens the effectiveness and pertinence of the literature and exacerbates the resistance to robotization.

Given that China is the world’s largest consumer market for robots, it is essential to conduct empirical research using China as a case to understand the impact of robots on employment quality in emerging markets. The research on the influence of robots on the employment quality of Chinese youth and the countermeasures will help other developing countries explore ways to improve youth employment. Based on this background, this is the first paper comprehensively studying the impact of robotization on youth employment quality in emerging regions and assessing the effectiveness of multiple measures on raising employment quality.

This research employs individual data from the China Household Finance Survey (CHFS) to investigate the impact and mechanisms of robots on youth employment quality. We use and improve the composite indices of robotization previously employed in the literature. With fixed-effects regression, this study found that the increase in robots significantly reduced youth employment quality, which remained valid with instrumental variables and robustness checks. Individual heterogeneity analysis revealed that the negative impact of robots was mainly concentrated on males, low-educated youths, and young workers aged 26–35. Regional heterogeneity analysis found that the impact of robots was mainly observed in regions with insufficient workers. Mechanism analysis revealed that the substitution effect, skill preparation effect, and productivity effect played essential roles in robot impact. An increase in regional education investment was found to significantly improve the employment quality of middle-educated youths, while personal skill improvement had a broader impact, with a significant positive effect on low-educated and middle-educated youths.

Compared with previous studies, the marginal contribution of this paper is reflected in the following aspects: Firstly, this research has solved an important but not fully elucidated question: whether the theory of SBTC is suitable for young people in developing countries? The answer to this question could help us better explain why people are increasingly wary of robotization even if the demand of employment market is still huge. We argue that the contribution of education is not dramatically decline, but the content of lessons for young people should focus on professional skill in developing countries.

Secondly, this research introduces a new perspective of employment quality to discuss the impact of robotization. Most existing studies have emphasized the impact of robots on job numbers [2,8], social inequality [9,10], and productivity [3]. However, the employment quality has not been given much attention in the empirical literatures, which is supplement to this field by this study. This study found that robotization has a negative impact on employment, especially on employment quality. It reminds scholars of focusing on the employment quality, not just the quantity of jobs, to avoid unhealthy and extensive growth. Thirdly, to better answer the research question, the mechanisms of robotization’s impact on employment quality is further studied. Previous studies have suggested many potential employment effects of robots, including the substitution effect [11], productivity effect [12,13], reinstatement effect [14], and structure effect [15,16], but we still lack a clear understanding of which effect works on youth employment quality. Based on the analysis framework of previous studies [11], this research confirms that the substitution effect, skill preparation effect, and productivity effect play a significant role, while the mediation effect of job creation is not significant, indicating that promoting the skill training of youth and the creation of new jobs should be the focus of employment policies.

Moreover, we provide a new and targeted way to youth for self-improvement and coping with robotization, In contrast to much literature that mainly focuses on the overall changes in employment [2,17], with inevitable neglect of the micro characteristics of some labor groups, this research focuses on the youth group, which makes the discussion of coping strategies more specific. Previous literature on this topic has emphasized the effects of education [7,18,19], unemployment training [20], and the industry allocation structure of robots [21]. In our work, we show that skill improvement can raise youth employment quality, and its impact range is even greater than that of education. Different from the conclusion that workers with higher levels of education profit most from training in developed regions [20], we find that in emerging regions, skill improvement is more effective for low- and middle-education youth. Given the high costs, long construction time, and slow effect of education enhancement, the discussion of other youth self-development measures is warranted.

The rest of this paper is organized as follows: the Literatures reviews section reviews relevant literature. The Research Design section describes the data and methods used in this paper. The Empirical results section presents the empirical results of the link between robotization and youth employment quality. Concluding remarks are summarized in the Discussion and conclusion section.

## Literatures reviews

The literature on how robotization affects employment and earnings is expanding but is still far from reaching a consensus, perhaps because of the differences in the national development level. The SBTC and task-model views respectively suggest that technological innovation increases unemployment risk and income polarization by reinforcing job skill differences [7] and altering job content [18]. In response, the theory of SBTC argues that highly educated labor is more suitable for the industrial demand in the future [22]. Some research on the employment effects of robots in developed countries such as the United States and Austria provide data to support this negative view [20,23]. However, it seems to change when differences in national development are taken into account, as in the innovative study of Graetz and Michaels (2018), which revealed a favorable impact on earnings and a neutral impact on employment by robotization [3]. Antón et al. (2022) found that the direction of robots’ impact on employment is uncertain and depends to a great extent on the model specifications and the selection of countries [24]. In contrast to existing evidence on advanced economies, a working paper of the World Bank shows a positive impact of robots on manufacturing and service employment in Indonesia [25]. These studies reveal national differences in the impact of robotization, underscoring the significance of re-testing in developing countries.

Robotization may put more pressure on young people. Technical change is destroying unskilled jobs, most especially the traditional “entry jobs”, deprived of jobs that could be filled by youngsters with little education and no particular skill or training [6], making youth employment face greater challenges [7,26]. Different from the theory of SBTC that highlight education [22], The research of Audu et al. (2013) emphasizes the significance of skill training to improve youth employment in developing countries without comparing it with other measures [27]. Simply and indiscriminately advising young people to pursue education or skill enhancement is more like a dubious labor economistic mantra than explicit career guidance [28]. Our study is therefore the first to compare the effectiveness of various measures to ameliorate technical unemployment among youth in developing countries.

Most previous research on robotization has focused on quantitative changes in employment [2,8], with a smaller number looking at employment quality in non-monetary forms, such as health and work intensity [29,30]. Closer to our topic is a recent paper by Antón et al. (2020). Antón et al. (2020) found that robotization significantly improved work intensity but had no effect on the physical environment, skills or discretion [4]. Nevertheless, it is worth mentioning that their data did not allow them to examine the effect of robotization on wage to fully assess the possibility that the high salary compensates for other deficiencies. Given that salary and working hours are central considerations in job selection, we will focus on hourly wage to examine changes in job quality, to complement previous research.

In the literature, there are four paths for the impact of robotization on employment: substitution effect, skill preparation effect, productivity effect, and job creation effect. The substitution effect refers to the fact that robots have a cost-comparative advantage over workers, and the risk of workers being replaced by robots increases sharply, significantly reducing workers’ employment advantage and wage bargaining space [12,14]. The skill preparation effect refers to the fact that workers may need to invest extra time and effort to learn and master robot-related skills [4,31], or to debug and maintain the robot to ensure its proper operation. The productivity effect refers to the fact that robotization does not necessarily increase short-term productivity [13], but the resulting changes in employment structure and redistribution of workers are likely to interrupt skill accumulation [32], as well as bring higher work intensity [4]. The job creation effect refers to the increased demand for workers for new jobs and tasks created by robotization. The job creation effect can compensate for the reduction in the number of jobs due to robotics to some extent [14,33]. We will empirically analyze whether these effects have an impact on the quality of youth employment.

**Fig 1:**
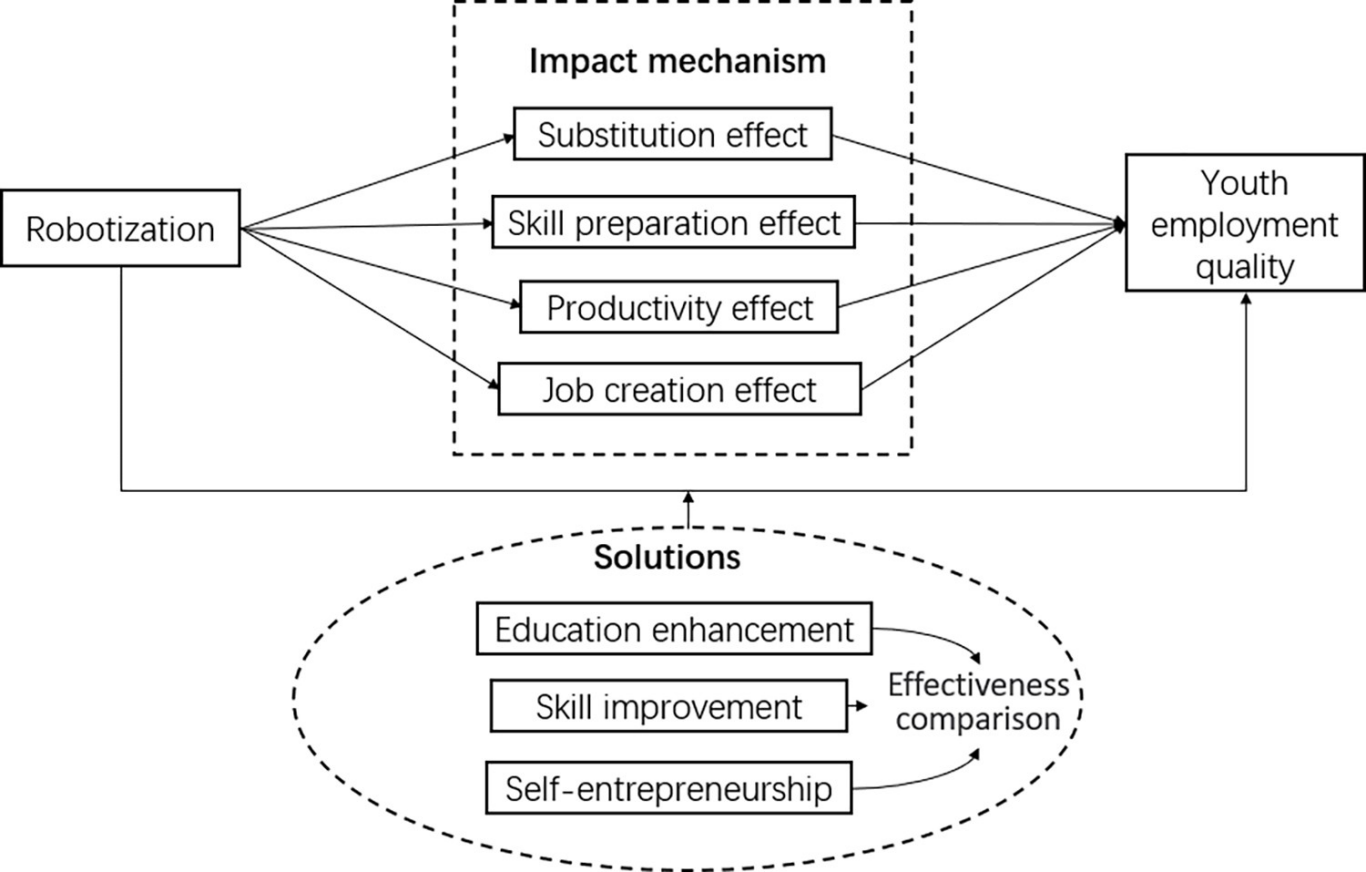
Research model.

Based on the preceding discussion, we construct the study’s theoretical model (Fig 1).

## Research design

### Econometric model setting and variable description

Based on the research question, the basic regression set in this paper is as follows:

$${Quality}_{ijt}=\alpha_{0}+\alpha_{1}{Robot}_{jt}+\gamma X_{ijt}+\mu_{t}+\varphi_{j}+\varepsilon_{ijt}$$ Eq (1)

*Quality*_*ijt*_ indicates comprehensive index of employment quality of individual *i* in province *j* in year *t*, *Robot*_*jt*_ measures the degree of robot use in province *j* in year *t*, *X*_*ijt*_ indicates control variables, *μ*_*t*_ for the year fixed effect, *φ*_*j*_ for the province fixed effect, *ε*_*ijt*_ is the residual term.

The explained variable measures the job quality of individual young workers. Existing literature typically uses three methods to measure job quality: one is the amount of wage received per unit of time [34], the second is job stability [15], and the third is the working environment [4]. Considering that the sample did not include information about the working environment, this research will use the first method to measure work quality and the second method for robustness testing.

In this study, robotization is defined the degree to which tasks in production and operations are accomplished through a combination of mechanical hardware, information, and data [35]. That means robotization includes not only robotic arms that perform simple tasks but also collaborative robots that monitor production and automatically adjust equipment parameters. As a result, the degrees of collection and usage of data are important indicators of robotization [5]. In addition, the economic and innovate benefits also are indispensable indicators for measuring robotization, because the benefits generated by current robotization will influence subsequent robotization decisions. Therefore, the measurement of robotization needs to consider hardware, infrastructure, and benefits.

The existing literature usually uses two ways to measure the degree of robot use. One is to establish a comprehensive index system of the economic and social benefits of robot use [35–37]; the other is to match IFR’s data with national industry data [38]. In this research, an index system of robot use degree is constructed by combining these two methods. The core explanatory variable in the model is the degree of robot use in each province. Due to lack of availability of more detailed data, we are unable to measure robotization more accurately. Nevertheless, the province dimension of explanatory variable does not affect the management connotation of the conclusions for two reasons. On the one hand, while decisions related to robotization are made independently, they are often influenced by the competitive advantages of nearby preceding robotized enterprises, resulting in that the level of robotization in the same region is likely to at a similar stage. On the other hand, it could provide a more holistic perspective by integrating micro and macro dimensions, avoiding lack of individual characteristics or market trends.

To sum up, our index system includes three parts: infrastructure construction, application in production, competitiveness, and benefit. With the principal component analysis, we get a comprehensive index. The specific index selection and explanation are shown in Table 1. The raw data of each indicator can be seen in S1 Dataset.

**Table 1:** Index system of the degree of robots use.

Composite indicator	Basic indicators	Sub-index	Index definition
Robot usage	Infrastructure construction	Software popularity and application	Revenue of basic software, supporting software, and application software divided by the main business revenue of industrial enterprises above the designated scale
		Input of intelligent equipment	Imports of computers, electronic components, instruments, and equipment divided by the main business revenue of industrial enterprises above the designated scale
		Information resource collection	The proportion of post and telecommunications business volume to GDP
		Data storage and processing	Consulting services revenue and operational services revenue divided by main business revenue of industrial enterprises above the designated scale
	Application in production	Application of industrial robots	The application of robots in industry. See below for specific calculations
		Production of new products	New product sales revenue divided by the main business revenue of industrial enterprises above the designated scale
		Platform operation and maintenance	Platform operation and maintenance service revenue divided by the main business revenue of industrial enterprises above the designated scale
	Competitiveness and benefit	Innovation capability	National patent application authorization divided by R&D personnel full-time equivalent
		Economic benefits	The total profit of industrial enterprises
		Social benefits	Electricity and coal consumption per unit of GDP

As for the use of industrial robots in the sub-index, the information provided by IFR is the total number of robots in each industry every year. The installation density of robots in each city cannot be directly obtained. Therefore, we refer to the existing document offering a method to construct a variable to measure the degree of robots at the provincial level [39]. The calculation formula is as follows:

$${Robot}_{jt}=\sum_{j=1}^{4}\left(E_{jyt}\times {Rob}_{yt}\right)/{emp}_{jt}$$ Eq (2)

*Rob*_*yt*_ is regional density of robots of province *j* in industry y at time *t*. *E*_*jyt*_ represents the share of the number of employees in industry *y* in year *t* in province *j* in the total number of employees in industry *y* in year *t* in the country. The parameter *Rob* is the number of robots installed in year *t* in industry *y* nationwide, and *emp*_*jt*_ is the number of manufacturing employees in year *t* in province *j*. Since the industry classification provided by IFR is not completely consistent with China’s labor industry classification, we refer to the existing methods [38] and select the four industries with the largest proportion of industrial robots in China among the industries with the same classification of manufacturing industry. This includes the automobile industry (51.6%), the electronic equipment industry (13.8%), the plastics and chemicals industry (11.2%), and the metals industry (5.0%).

The control variables in this research include individual socio-demographic characteristics and province-level characteristics. Individual characteristics include age, gender, marital status, and health conditions. Province-level characteristics include economic development level, industrial structure, and average employment costs.

### Data source and descriptive statistics

In this study, socio-demographic characteristics (age, gender, health status, marital status) and employment information (wage, working hours, work stability) were obtained from the 2017 and 2019 CHFS. The rest of the data came from IFR’s industrial robot installation data, the China Statistical Yearbook, the China electronic information industry statistical yearbook, and the China Stock Market and Accounting Research Database.

The obtained data is processed as follows: firstly, to focus the study on employment quality, samples with a value of 0 for working hours or wages are removed. Secondly, considering the greater pressure of robotization that young people may face, samples younger than 17 years old or older than 35 years old are excluded. Finally, samples with missing other key data are eliminated. After the above data processing, we obtained 13453 samples that are distributed across 29 provinces, except for Tibet, Xinjiang, Hong Kong, Macau, and Taiwan. A 99% tail truncation is utilized to ensure that extreme values does not unduly affect the results. Table 2 presents the definitions and summary statistics of the variables. Overall, all the data used in this study has been uploaded for sharing as the S2 Dataset of this article.

**Table 2:** Variable definitions and basic statistics.

variable	Definition	N	mean	sd	min	max
Quality	Wages paid on an hourly basis	13453	51.92	105.3	3.333	649.4
Robot	A comprehensive index calculated from the aspects of infrastructure construction, production application, competitiveness, and efficiency	13453	2.399	3.498	0.564	19.84
age	Year of survey minus year of birth	13453	28.58	4.263	17	35
gender	Man = 1, women = 0	13453	0.577	0.494	0	1
marriage	Marital status (Married = 1, unmarried = 0)	13453	0.555	0.497	0	1
health	Self-rated health status (healthy = 1, unhealthy = 0)	13453	0.974	0.158	0	1
lnGDP	Logarithm of regional GDP	13453	10.26	0.827	7.873	11.59
structure	The ratio of the third sector to the second sector	13453	1.383	0.698	0.852	5.169
lnaverwage	Logarithm of average wages	13453	11.21	0.213	10.92	11.91

## Empirical results

### Baseline model results

We used the OLS mixed regression, the fixed effects model, and the random effects model to estimate our regression. To avoid bias caused by too few clusters of independent variables, we use the robust standard error, clustered by individual of the dependent variable, throughout. This is a general way to deal with too few clusters of independent variables.

Table 3 reports the impact of robotic use on the quality of youth employment. Column (1) presents the results of a mixed-effects OLS regression controlling for year and region. Column (2) shows the fixed-effects regression results, and column (3) presents the random-effects regression results. From the second and third columns, the presence of robots has a significant negative impact on the quality of youth employment, whether considering fixed effects or random effects. Based on the Hausman test result, we mainly focus on the fixed-effects regression results. The results show that when other variables are fixed, when the independent variable Robot increases by one unit, the value of the dependent variable youth employment quality will decrease by 5.172.

**Table 3:** Results for the linear model analysis.

VARIABLES		Quality	
	(1)	(2)	(3)
Robot	-4.844***	-5.172**	-4.844***
	(1.348)	(2.605)	(1.348)
age	0.673***	2.762	0.673***
	(0.160)	(13.287)	(0.160)
gender	7.000***	-67.575	7.000***
	(1.043)	(99.150)	(1.043)
marriage	-0.860	54.335	-0.860
	(1.368)	(33.183)	(1.368)
health	7.396*	-11.006	7.396*
	(3.825)	(40.454)	(3.825)
lnGDP	113.529***	143.989***	113.529***
	(28.762)	(53.832)	(28.762)
structure	77.184***	101.269**	77.184***
	(21.928)	(44.038)	(21.928)
lnaverwage	68.307	-500.106	68.307
	(183.826)	(345.134)	(183.826)
Region FE	Yes	Yes	Yes
Year FE	Yes	Yes	Yes
Individual FE	No	Yes	No
N	13,453	13,453	13,453
Hausman Test		0.000	
R^2^	0.631	0.725	-

Robust standard errors, clustered by individual, are in parentheses. Significance levels are represented as

*** p<0.01

** p<0.05

* p<0a.1.

The regression results confirm that the use of robots will have a negative impact on the quality of youth employment. With the widespread promotion of machine substitution by national and local governments, the density of robots in cities is rapidly increasing, and the requirements for the skills of young workers will become increasingly higher. Therefore, it is necessary to focus on the downward trend in youth employment quality and possible measures to address it.

It is noted that some control variables are not significant in the regression, although they do not affect the significance of the main effect. The potential reason may be that limiting factors like incongruous proportions of the sample in terms of gender and age obscure underlying correlations [40–42]. We refer to the treatment of Brall and Schmid (2023) and conduct a classification discussion based on individual heterogeneity [43], to further analyze the individual heterogeneity of robotization effect.

### Endogeneity problem and robustness test

To address potential endogeneity issues, this article adopts a practice found in existing literature [44] and uses the optical fiber density and average electricity consumption of the employed population as instrumental variables. Increasing the use of robots will increase the demand for data transmission, communication, control, and monitoring, while optical fiber density will not have a direct impact on the quality of employment for young people. Similarly, the increased use of robots may lead to a large increase in electricity consumption, which may also be caused by factory expansion. Therefore, we estimate electricity use divided by employment as another instrumental variable.

The two-stage least squares estimation results using instrumental variables are shown in Table 4. The results in column (2) show that the KP Wald-F is 32.855, which is higher than the 10% bias threshold (19.93), and there is a significant correlation between the independent variable and the two instrumental variables, indicating that the instrumental variable can pass the weak instrumental variable test. The result of LM test is 0.000 (<0.05), proving that the instrumental variable can pass the under-identification test. In addition, the results of the exclusion test showed that the value of Hansen J is 0.053 (>0.05), indicating that the instrumental variable can pass the exclusion restriction test (over-identification test). The results in column (1) show that under the test of the instrumental variable method, the impact of robotization on the employment quality of Chinese young people is still significant and negative.

**Table 4:** Results of endogeneity test and robustness test.

VARIABLES	The second stage	The first stage	Explanatory variable substitution	Dependent variable substitution		Control variables increase
	Quality	Robot	Quality	stability	Quality	Quality
	(1)	(2)	(3)	(4)	(5)	(5)
Robot	-60.639***			-0.015**	-0.023***	-5.223**
	(18.372)			(0.007)	(0.007)	(2.587)
IV: optical fiber density		1.278***				
Robot		(0.207)				
IV: Average electricity consumption of employed population		1.705***				
Robot		(0.229)				
Installation			-0.038**			
			(0.016)			
KP Wald-F		32.855				
Threshold		19.93				
LM Test		0.000				
Hansen J		0.053				
Region FE	Yes	Yes	Yes	Yes	Yes	Yes
Year FE	Yes	Yes	Yes	Yes	Yes	Yes
Individual FE	Yes	Yes	Yes	Yes	Yes	Yes
N	13,453	13,453	13,453	13,453	13,453	13,453
R^2^	-	-	0.726	0.407	0.486	0.725

Robust standard errors, clustered by individual, are in parentheses. Significance levels are represented as

*** p<0.01

** p<0.05

* p<0.1.

Additionally, we conducted robustness analyses in three aspects. Firstly, we replaced the measure of robot density with a new variable that allocated the annual installation amount of robots according to the share of the employment population in each province in the national territory. Secondly, we replaced the indicator of the explained variable with job stability, a binary variable depending on whether the employment contract is signed. Thirdly, we replaced the dependent variable with a composite indicator that combines wage (after normalization) and job stability. Finally, we added a new control variable to the model. Since ownership of the employer may affect the salary and work intensity, we generated virtual variables for ownership of the employer based on public institutions, public or collective enterprises, joint ventures, self-employed individuals, private enterprises, and others, and included them in the model for re-regression.

From the results in Table 4, the coefficient of the core explanatory variable is still significantly positive after replacing the explanatory variables (see column (3)), replacing the explained variable (see column (4–5)), and adding control variables (see column (6)), indicating that the negative impact of robotization on youth employment quality has successfully passed the robustness test. The conclusion of this study is reliable.

### Heterogeneous analysis

To investigate whether there are heterogeneity effects of robots on youth employment quality, we conducted individual heterogeneity analyses on the sample based on gender, education level, and age. Educational attainment was divided into primary school, middle school (including junior high school and high school), and college groups. The results are reported in columns (1) to (7) of Table 5.

**Table 5:** Heterogeneity analysis results.

VARIABLES	Individual heterogeneity							Regional heterogeneity	
	Gender		Education level			Age		employment-population proportion	
	Man	Women	Primary	Middle	university	17–25	26–35	Large	Small
	(1)	(2)	(3)	(4)	(5)	(6)	(7)	(8)	(9)
Robot	-5.55*	-3.43	-1.00	-6.72**	53.77	2.34	-5.41*	-1.18	-48.42***
	(3.21)	(3.70)	(3.20)	(3.36)	(53.96)	(5.86)	(2.82)	(2.73)	(17.17)
Region FE	Yes	Yes	Yes	Yes	Yes	Yes	Yes	Yes	Yes
Year FE	Yes	Yes	Yes	Yes	Yes	Yes	Yes	Yes	Yes
Individual FE	Yes	Yes	Yes	Yes	Yes	Yes	Yes	Yes	Yes
N	7,760	5,693	5,453	7,556	444	3,366	10,087	4,818	8,635
R^2^	0.72	0.78	0.76	0.75	0.90	0.78	0.73	0.76	0.74

Robust standard errors, clustered by individual, are in parentheses. Significance levels are represented as

*** p<0.01

** p<0.05

* p<0.1.

Firstly, robots have a negative impact on the employment quality of male youth, while there is no significant impact on female youth. This may be attributed to gender-based innate physiological differences, resulting in a wider distribution of male workers in labor-intensive jobs that often involve repetitive tasks, which are precisely the areas where the substitution effect is severe.

Secondly, robots have a significant negative impact on the employment quality for the medium-educated group, while the high-educated youth and the low-educated youth do not show a significant impact. This conclusion is consistent with some existing studies [45], which found that automation has reversed the demand for labor at the high end of the labor market, reducing the demand for medium-educated labor, represented by high school and middle school labor. The reason may be that the wages of low-educated workers are relatively low, and robots do not have a significant cost advantage compared to them. High-educated groups mainly engage in creative work, which is less likely to be replaced by existing automation technology. Medium-educated groups mainly perform tasks with low technological content, and their ability to respond to machine impacts is also relatively low, making their job more vulnerable to being weakened by robotization.

Finally, those who are aged between 26 to 35 are more significantly affected by the negative impact of robots than those who are aged 17 to 25. This may be because the rapid increase in graduates in recent years has changed the supply and demand environment of the youth job market, and adolescents need to complete the transformation of their thinking and abilities from school to work. If young workers do not accumulate enough skills in their transition period, they are likely to be replaced by robots or more active and younger workers.

In addition, we also conducted a regional heterogeneity analysis, dividing the sample into two groups based on whether the proportion of the employed population among regional populations is higher than the average, to measure the difficulty of obtaining labor. The results are reported in columns (8) and (9) of Table 5.

The regression results of regional heterogeneity show that the negative impact of robots on youth employment quality is more significant in low-proportion areas of employed population, while it is not significant in high-proportion areas. In areas with a low proportion of employed people, the difficulty of obtaining labor that meets skill requirements is higher, and enterprises are more motivated to use robots to complete work, further squeezing the survival space of workers in these areas. In areas with a high employed population proportion, enterprises have greater hiring options and can hire workers at a relatively reasonable price in fierce employment competition, so they are not eager to introduce robots.

### Mechanism analysis

To further understand the mechanism of the impact of robotics on the employment quality of young workers, we have verified the “substitution effect”, “skill preparation effect”, “productivity effect” and “job creation effect”. We construct the following model for mechanism analysis:

$$N_{ijt}=\beta_{0}+\beta_{1}{Robot}_{jt}+\gamma X_{ijt}+\mu_{t}+\varphi_{j}+\varepsilon_{ijt}$$ Eq (3)

$${Quality}_{ijt}=\alpha_{0}+\alpha_{1}{Robot}_{jt}+\alpha_{2}M_{jt}+\gamma X_{ijt}+\mu_{t}+\varphi_{j}+\varepsilon_{ijt}$$ Eq (4)

For the substitution effect and skill preparedness effect, we constructed Eq (3) to test them, where *N*_*ijt*_ represents monthly income and monthly work hours. For the productivity effect and job creation effect, we used the bootstrap sampling method (1000 times) to test the mediation effect of the productivity effect and job creation effect on the impact of robots on youth employment quality and constructed Eq (4) to test the degree of mediation effect, where *M*_*jt*_ represents regional productivity and employment population.

Table 6 reports the empirical results of the internal mechanism test. The results in columns (1) and (2) show that robots have a significant negative impact on the salary of young employees and significantly increase their working hours, confirming the substitution effect and skill preparation effect of robots on youth employment. The increase in robots does not have a significant impact on regional productivity, but the productivity effect has a significant negative impact on youth employment quality.

**Table 6:** Results of mechanism analysis.

VARIABLES	wage	workhours	Quality	Quality
	(1)	(2)	(3)	(4)
Robot	-845.991*	2.109***	-5.137**	-6.581**
	(461.365)	(0.744)	(2.557)	(2.678)
productivity			-843.598*	
			(491.665)	
workers				0.237*
				(0.143)
Bootstrap			[0.490, 0.035]	[-0.305, 0.576]
Region FE	Yes	Yes	Yes	Yes
Year FE	Yes	Yes	Yes	Yes
Individual FE	Yes	Yes	Yes	Yes
N	13,453	13,453	13,453	13,453
R^2^	0.757	0.046	0.727	0.727

Robust standard errors, clustered by individual, are in parentheses. Significance levels are represented as

*** p<0.01

** p<0.05

* p<0.1.

The result of the bootstrap sampling method in column (3) shows that the confidence interval of the indirect effect does not include 0, indicating the productivity effect plays a mediation role in the impact of robots on youth employment quality. With the mediator variables controlled, the impact of robots on youth employment quality remains significant, indicating that the productivity effect plays a partial mediation role and has a significant negative impact on youth employment quality. This result responds to the previous study [14,32], which suggests that the negative own-industry employment effect of rising productivity has not diminished aggregate labor demand but has resulted in skill-biased demand shifts. However, the industrial shifts deprive young workers of the opportunity to deepen their skills in their own industry and force them to be shifted to another industry where they do not have experience or advantages. Additionally, the uneven distribution of robots offers another possible explanation [21]. The introduction of robots in China may have only occurred in a few manufacturing enterprises with a low share of labor in firm value-added, bringing higher market concentration and even intensified monopoly rather than productivity improvements in the entire region.

Although the coefficient of the job creation effect is significant, the Bootstrap sampling results show that the job creation effect does not play a mediating role in the impact of robots on youth employment quality. This result supplements an existing study [12], which found that the introduction and expansion of new tasks and job titles explain approximately half of US employment growth from 1980 to 2010. The job creation effect has a positive impact on total employment, while its impact on employment quality is not significant. This may be because China’s robot application has not yet fully penetrated all production processes and the driving power of industrial transformation and upgrading is still insufficient, resulting in a limited number of new jobs.

### Regulatory effect analysis

To address the potential employment issues brought about by robots, it is necessary to develop appropriate strategies and evaluate their effectiveness. We included several common strategies for employment issues in the regression, including education enhancement, skill improvement, and self-entrepreneurship. Specifically, we measured education enhancement by the proportion of public expenditure on education relative to total public expenditure, skill improvement by the number of people who obtained professional certification qualifications, and self-entrepreneurship by the question "At present, does your family engage in commercial and industrial production and trading projects?" from the CHFS. The cross-multiplication terms of the independent variable (*Robot*) these and three strategies are respectively included in the regression as the regulating variables. If the coefficient of the cross-multiplication term is significantly positive, it means that the strategy can effectively alleviate the negative impact of robotization on youth employment. The results are presented in Table 7.

**Table 7:** Results of regulatory effect analysis.

VARIABLES		Quality	
	(1)	(2)	(3)
Robot	-23.5211***	-23.0931***	-5.5624**
	(7.5345)	(7.7000)	(2.5907)
Robot×edu	830.7475**		
	(325.3017)		
Robot×skill		64.9515**	
		(26.4707)	
Robot×entrepreneurship			-8.5957
			(6.7868)
Region FE	Yes	Yes	Yes
Year FE	Yes	Yes	Yes
Individual FE	Yes	Yes	Yes
N	13,453	13,453	13,453
R^2^	0.7301	0.7293	0.7255

Robust standard errors, clustered by individual, are in parentheses. Significance levels are represented as

*** p<0.01

** p<0.05

* p<0.1.

The results show that education enhancement has a significant positive regulatory effect on youth employment quality, indicating that increasing investment in education can indeed help improve future employment conditions. It is worth noting that skill improvement has a significant positive role in improving youth employment quality, which provides a new way for improving young people’s employment. However, the impact of self-entrepreneurship is not significant, possibly because it involves high risk and requires a sufficient understanding of a particular industry and financial support, which are precisely the things that young people lack.

Table 8 shows the results of the effectiveness analysis of education enhancement and skill improvement, conducted by dividing the sample into three groups based on educational attainment. It can be found that education enhancement has a significant positive impact on the middle-level education group, but there is no significant positive impact on the low-educated group or high-educated education group. These indicate that China faces a pressing need to strengthen secondary education and improve the educational attainment of the middle-level education group. Compared to education enhancement, the impact coefficient of skill improvement is not high, but its impact range is wider. Skill improvement has a significant positive regulatory effect on the low-level education group and the middle-level education group, indicating that the government needs to build places suitable for young people to learn and practice professional skills. This also provides empirical support for the vocational education classification policy, which aims to improve the employment situation of young people through two paths: knowledge education and skill training.

**Table 8:** Results of the effectiveness analysis of employment promotion measures.

VARIABLES	degree of education					
	Low	Middle	High	Low	Middle	High
	(1)	(2)	(3)	(4)	(5)	(6)
Robot	-5.703	-30.996***	90.397**	-29.998**	-30.561***	77.234***
	(12.523)	(8.438)	(35.276)	(12.135)	(8.768)	(26.456)
Robot×edu	211.069	1,127.640***	-2,441.029**			
	(557.425)	(362.407)	(1,012.366)			
Robot×skill				104.718**	88.571***	-363.445***
				(42.783)	(29.876)	(81.774)
Region FE	Yes	Yes	Yes	Yes	Yes	Yes
Year FE	Yes	Yes	Yes	Yes	Yes	Yes
Individual FE	Yes	Yes	Yes	Yes	Yes	Yes
N	5,453	7,556	444	5453	7,556	444
R^2^	0.757	0.763	0.912	0.772	0.761	0.924

Robust standard errors, clustered by individual, are in parentheses. Significance levels are represented as

*** p<0.01

** p<0.05

* p<0.1.

## Discussion and conclusion

The impact of advances in automation on employment and sustainable development has long been debated. In recent years, the rapid development of robot applications in emerging markets, represented by China, arouses academic interest in the impact of robotization on employment in developing countries. Compared with middle-aged employees, young employees are in the transition period from school to society and lack enough technological accumulation to strengthen their irreplaceable position. This raises a question: whether the theory of SBTC is suitable for young people in developing countries? While there are a relevant number of studies concerned with the impact of robots and related technologies on the number of jobs and work quality in non-monetary terms, our research contributes to understand the problem from a monetary perspective. In addition, our study is the first quantitative analysis of how youth can improve in the context of robotization, which is more targeted than vague recommendations and will have implications for other emerging markets dealing with the employment impact of technology.

We employ data from the 2017 and 2019 Chinese Household Finance Survey and the International Federation of Robotics to explore how the adoption of robots affects youth employment quality in emerging markets. After endogeneity and robustness test, this study conducts individual and regional heterogeneity analysis. Base on the exploration of mechanism, three countermeasures are employed as regulatory variables to further explore which ways are effective for youth to maintain and improve employment quality.

In response to the research question, we conclude that robotization has a negative impact on the employment quality of Chinese youth according to the results. This influence is more significant for male youth, young people who are middle-educated and aged 26 to 35, and youth in labor-shortage areas. The substitution effect, skill preparation effect, and productivity effect play important roles in this process, while the job creation effect did not significantly improve youth employment quality.

We found that education is still effective in enhancing employment competitiveness in the background of robotization, which supports the theory of SBTC. Furthermore, professional skill upgrading has a wider impact range than universal education enhancement and has a positive effect on the employment quality of both low-educated youth and medium-educated youth. That suggests that the curriculum for young people needs to reformed to incorporate more skill training.

This study suggests that robotization may not only have a potential impact on job numbers but also potentially affect employment quality, especially for young workers who lack skill accumulation. In contrast to the popular emphasis on job numbers, the real danger for young workers may not come from changes in job numbers but from the reduction in employment quality because robots are not sufficiently productive to generate enough high-quality jobs. This study also confirms that professional skill training can guide youth to better cooperate with robots and engage in professions that are difficult to automate, which provides new ways for youth to deal with the employment impact of robots besides increasing education investment. In fact, the vocational education classification policy in China is a meaningful attempt to alleviate the dilemma of scarce higher education resources and insufficient skilled talent. Improving the situation of youth employment through two paths—knowledge education and skill training—may be the mainstream trend for future employment in emerging markets.

There are some areas to be improved in this study. On the one hand, employing different dimensions of measure of robotization for duplicated test is beneficial to improve the reliability of conclusions. The imbalance in individual characteristics of samples also needs to be further analyzed. We look forward to the publication of more detailed and diverse data to support research about the impact of robotization. Besides, a comprehensive indicator that integrate more dimensions of employment quality needs to be proposed urgently. On the other hand, more diverse approaches need to be developed to fully understand the impact of robotization on youth employment quality. For example, the difference in difference analysis according to the landmark event of robotization can be used to verify and supplement the research conclusion of this study from another perspective.

## Supporting information

S1 Dataset The raw data of each indicator of robotization. (XLSX)

S2 Dataset The whole dataset for this study. (XLSX)
